# Total Economic Value of Wetlands Products and Services in Uganda

**DOI:** 10.1155/2013/192656

**Published:** 2013-09-15

**Authors:** Willy Kakuru, Nelson Turyahabwe, Johnny Mugisha

**Affiliations:** ^1^School of Forestry, Environment and Geographical Sciences, Makerere University, Kampala, Uganda; ^2^Department of Extension and Innovation Studies, College of Agricultural and Environmental Sciences, Makerere University, Kampala, Uganda; ^3^Department of Agribusiness and Natural Resources, College of Agricultural and Environmental Sciences, Makerere University, Kampala, Uganda

## Abstract

Wetlands provide food and non-food products that contribute to income and food security in Uganda. This study determined the economic value of wetland resources and their contribution to food security in the three agroecological zones of Uganda. The values of wetland resources were estimated using primary and secondary data. Market price, Productivity, and Contingent valuation methods were used to estimate the value of wetland resources. The per capita value of fish was approximately US$ 0.49 person^−1^. Fish spawning was valued at approximately US$ 363,815 year^−1^, livestock pastures at US$ 4.24 million, domestic water use at US$ 34 million year^−1^, and the gross annual value added by wetlands to milk production at US$ 1.22 million. Flood control was valued at approximately US$ 1,702,934,880 hectare^−1^ year^−1^ and water regulation and recharge at US$ 7,056,360 hectare^−1^ year^−1^. Through provision of grass for mulching, wetlands were estimated to contribute to US$ 8.65 million annually. The annual contribution of non-use values was estimated in the range of US$ 7.1 million for water recharge and regulation and to US$ 1.7 billion for flood control. Thus, resource investment for wetlands conservation is economically justified to create incentives for continued benefits.

## 1. Introduction

Wetlands provide important natural resources, upon which the rural economy in Eastern Africa depends [1]. They provide many substantial benefits not only to local society, but also to the people who live far away from them. They are recognised globally for their vital role in sustaining a wide array of biodiversity and providing goods and services [2] and also as important sources of natural resources, upon which the rural economies depends [3].

In Uganda, wetlands provide a wide range of tangible and nontangible benefits to various communities [4, 5]. The tangible benefits include water for domestic use and watering of livestock, support to dry season agriculture, provision of handicrafts, building materials, and food resources such as fish, yams, vegetables, wild game, and medicine. The non-tangible benefits include flood control, purification of water, maintenance of the water table, microclimate moderation, and storm protection. Wetlands also serve as habitats for important flora and fauna, have aesthetic and heritage values, and contain stocks of biodiversity of potentially high pharmaceutical value [4, 5]. All these benefits have a bearing on food security.

Over 80% of the people living adjacent to wetland areas in Uganda directly use wetland resources for their household food security needs [6]. Besides, they also indirectly contribute to food security by providing services that foster food production such as weather modifications and nutrient retention. Food security exists when all people, at all times, have physical and economic access to sufficient, safe, and nutritious food that meets their dietary needs and food preferences for an active and healthy life [7]. The dimensions of food security include availability, access, and utilisation. Wetland resources play a vital role in contributing to food security through the following:

- enabling direct availability of products such as fish, crops grown along the wetland edges, wild fruits and vegetables, and game meat;
- providing cash income from sale of raw materials and processed products such as crafts, sand, clay, bricks, and ecotourism; which are sold for cash that is used for purchasing/accessing food; and
- contributing to increased crop and livestock yields as a result of improved productivity from use of water, silt, and through climate moderation.

Each of the individual benefits or attributes of wetlands contributes to the household's output, welfare, or utility, thus making wetlands a recognised enabling sector to the economy of Uganda [8]. However, some of the benefits are marketed and can be allocated for monetary values, while others are used at subsistence level and do not have a direct reflection of their monetary values. This often makes it difficult to prioritise allocation of resources for the management and conservation of wetlands. This has led to continued degradation and low economic value attached to sustainable wetland resources management. To guide decisions on wetland management options, it is important to express the benefits derived from wetland resources in quantified monetary terms, as the basis for economic valuation. Wetland economic valuation is defined as a way of attaching quantitative and monetary values to wetland goods and services, whether or not market prices are available, so that they can be directly comparable with other sectors of the economy when activities are planned, policies are formulated, and decisions are made [9]. A better understanding of the benefits and costs of utilising wetland resources will provide important information for understanding and addressing the economic causes of wetland degradation and loss. 

This study was undertaken to determine the economic values of wetland resources, to quantify wetland economic benefits and costs, to and determine the economic value of nonmarketed wetland goods and services in Uganda. The study highlights economic benefits in monetary terms for selected key wetland goods and services and demonstrates to wetland users, managers, and policy makers how valuable wetland resources are, as a basis for guiding decision making on wetland conservation.

## 2. Materials and Methods

### 2.1. The Study Area

**Figure 1:**
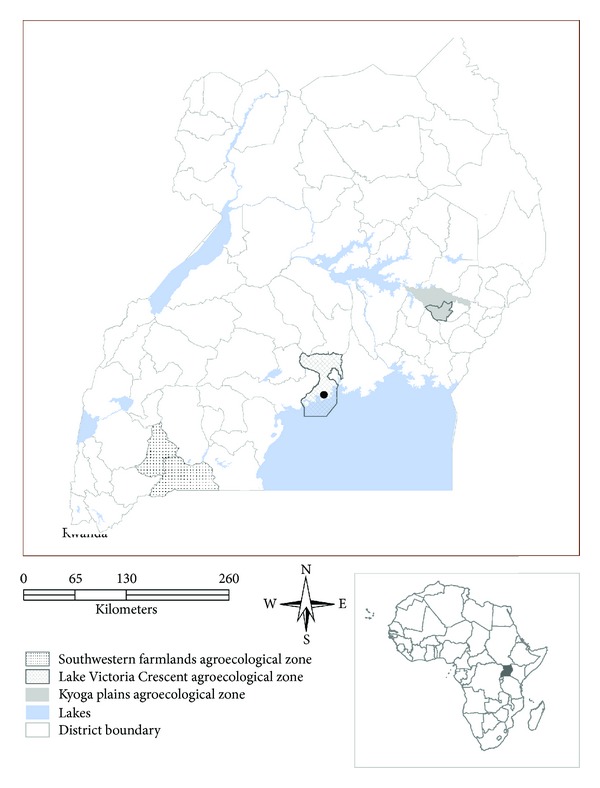
Map of Uganda showing the study sites.

The study was conducted in eight wetland systems located in areas that represent three of the five agroecological zones of Uganda. The wetlands are Nangabo, Mabamba, and Mende in Wakiso district representing the Lake Victoria crescent agroecological zone; Rucece in Mbarara and Lake Nakivale in Isingiro representing Southwestern farmlands; Limoto and Gogonyo in Pallisa and Kibuku Districts representing the Kyoga plains agroecological zone (Figure 1). These wetlands offer different benefits to local communities, have different biophysical characteristics, experience varied socioeconomic conditions and are faced with dissimilar management challenges. This study followed three methods for quantifying the monetary values of wetland services and goods, namely, the market price method [4, 9, 10], the productivity method [11, 12], and the contingent valuation method [11, 13, 14]. The market prices method was applied to quantify direct use values, by estimating the price in commercial markets for such wetland resources as papyrus products, pastures, and fish. The respondents made an estimate of the value of nonmarket goods by utilising direct surveys to solicit responses that reflect each individual resource user's valuation of a nonmarket good. The productivity method was used to quantify the use of water. The contingent valuation method was used for nonuse values such as flood attenuation, water recharge and supply, and habitat and breeding.

### 2.2. Data Collection and Computation of Wetland Values

Consultative meetings were held with environment and wetlands managers of the selected wetland areas to seek their opinions on the most important wetland resources to the communities, challenges, and opportunities for their sound management. Following discussion and advice from wetland managers, important wetland resources for valuation were selected based on (i) whether a resource met the basic needs of the communities from the study area; (ii) number of users harvesting the resource; (iii) whether the resource represented a range of uses to the different users; and (iv) the likelihood of obtaining sufficient quality data on the resource to enable computation of economic values. The other factor considered was whether harvesting, sale or use of the selected resource were particularly important or widespread or where it generated significant local benefits. A summary of the wetland resources selected for valuation is presented in Table 1.

Opportunistic sampling was made for respondents in areas where different wetland resources were harvested, processed, or marketed. Data were collected through interviewing at least 10 respondents for different resources on the value they attached to the wetland goods and services using structured questionnaire interviews. Focus group discussions were also conducted with different wetland resources users to generate information on the uses and associated costs of the resource under valuation. We also reviewed information from district inventory reports on the crop and animal production, population, prices of the associated goods, and wetland area coverage. Value transfers from previous studies [4, 10, 11, 13] were used to compute the values of wetland goods and services. 

Data on economic value of wetlands for crop farming in 2012 were collected by estimating the total farming area in each of the agroecological zone and the area of wetlands under crop production from the district inventory reports. We also collected data on the yields and the number of harvests per year for the key crops grown in the wetlands. The key crops considered were maize in the southwestern farmlands agroecological zone, vegetables in the Lake Victoria crescent, and rice in the Kyoga plains agroecological zone. 

To estimate the value of wetlands for fish breeding, data on the total spawning area were collected from the wetland coverage in the three agroecological zones following spatial data from the National Wetlands Information System. The data were used to derive the estimated value of wetlands for fish breeding per hectare per year. 

We also collected data on the percentage of the total number of livestock depending on wetlands and the average daily pasture consumption per animal to estimate the economic value of wetlands to food security through livestock production. Data on the total livestock numbers in the three agroecological zones were obtained from the Ministry of Agriculture, Animal Industry and Fisheries (MAAIF) reports. We only took a conservative estimate of the value of wetland pastures only for cattle, leaving out other types of livestock such as pigs, goats, and sheep. The cost of pastures was inferred from the imputed value of the cost of alternative leafy feeds that would be bought, if wetland pastures were not available, estimated at a cost of US$ 0.2 per animal per day following Karanja et al. [4]. Data were also collected on the number of cattle directly using water from wetlands and the daily consumption of water, which was imputed at 40 litres per animal per day. The value added through milk production was derived from data collected on the number of milked cows and the annual milk production in relation to the price of milk in the three agroecological zones.

The value of grass mulch was estimated using data on the total land area under banana production. Only two districts of Isingiro and Mbarara in the southwestern farmlands agroecological zone were considered in estimation of the economic value of grass mulch in bananas because this is only where the resource was used. Reports from the focus group discussions indicated that in the two districts, nearly every household was engaged in banana growing. It was estimated that on average each family in the two districts owned at least one hectare of bananas. This was supplemented by data on the total acreage of bananas that were mulched with wetland grass, estimated at 50% of banana plantations, and the number of bundles of mulch applied in the banana plantation per hectare per year.

The economic value of papyrus to food security through food accessibility was computed from data collected on the returns to papyrus resource users by either selling raw papyrus materials or after value addition through mat making. Estimates of the total wetland area in the district under papyrus and the productivity per hectare in head loads were made to generate the total productivity per annum. For the craft products from papyrus, data were collected on price of the raw material and of the products, labour costs for harvesting the product, equipment, additives, storage, licenses, transport, hired, and personal time to derive the net returns.

The economic value of wetlands for fresh water storage and supply was estimated by use of data collected on the number of household dependents on wetlands for water supply and annual water use for all the households. This was extrapolated to the midyear total human population projections of 2012. About 80% of the populations depend on wetlands for domestic water supply as used by Karanja et al. [4] and WMD et al. [5]. The contribution of wetland through nonuse values such as flood attenuation, water recharge and supply, and habitat provision was determined by use of value transfers following Karanja et al. [4] and Turpie [10].

Data on the management costs for conserving wetlands to reflect willingness to conserve (WTC), as reflected in the economic costs for wetland management and conservation, was generated from the existing costs incurred by districts in the wetland management sector. These included costs for staff salaries and allowances, equipment, and their maintenance and monitoring compliance to wetland conservation. Data on estimated income and other benefits foregone from land use, as well as investment and development opportunities precluded or diminished, to maintain wetlands were used to compute opportunity costs. National data from the Ministry of Water and Environment were used to compute the management and operation costs of conservation. In the three agroecological zones, wetland management and conservation were supported by remittances from the central government, Ministry of Water and Environment, and the locally generated revenue from the respective districts.

## 3. Results 

### 3.1. The Economic Value of Wetlands through Fish Breeding/Spawning and Availability

In terms of spawning habitats for fish, wetlands in Uganda contributed an estimated gross value of US$ 1,091,444 per year (Table 2). Wetlands do not only serve as breeding grounds for fish whose habitat is shallow waters, but were also mentioned as important spawning areas for fish that live in deep open water. On average, fish available for consumption from wetlands in the three agroecological zones of Uganda were equivalent to US$ 0.49 per person (Table 3). During the focus group discussions, fish was reported as a key source of less expensive animal protein, compared to chicken, beef, and goat meat.

### 3.2. The Economic Value of Wetlands through Crop Farming

The economic value of wetlands to crop farming was estimated to be in the range of US$ 417,536 to 25.09 million (Table 4). In all the three agroecological zones, wetland adjacent communities noted that yields from wetland crop farming were higher, owing to the moisture guaranteed even during the drought periods. This was in addition to fertility replenishment of the wetland ecosystem from sediment trapping and gradual settling of silt particles and rotting of organic matter from wetland vegetation. This is an indicator that guided use of wetland edges for crop farming largely contributes to livelihoods of surrounding communities and can provide incentives for their involvement in wetlands conservation.

### 3.3. The Economic Value of Wetlands from Grass Mulch

The gross annual contribution of the wetlands to food security, through provision of grass for mulching, was estimated at US$ 8.65 million per annum (Table 5). Wetlands provided grass mulch that enhanced crop productivity, particularly for banana production in the southwestern farmlands agroecological zone.

### 3.4. The Economic Value of Wetlands from Pastures and Water for Livestock

Wetlands provided livestock pastures worth US$ 4.24 million (Table 6). Focus group discussions revealed that wetlands were vital grazing areas during the drought periods, when alternative pastures were not readily available. The importance of wetlands was also more significant due to the fact that alternative livestock feeds were expensive and were not easily affordable by most farmers, as reported in the focus group discussions of this study.

The total economic value of water from wetland areas for livestock consumption was estimated to be worth US$ 34 million per year (Table 7). During focus group discussions, the wetlands were reported to serve as watering points not only for the wetland adjacent communities but also to distant livestock farmers. For most free range livestock grazing, the most common source of water for livestock in the study areas was wetlands.

The gross annual value of wetlands to milk production was estimated at US$ 1.22 million (Table 8). About 10% of the total production of milk in the study areas was attributed to grazing livestock within wetlands. During the focus group discussions, the respondents reported that wetlands are more vital during the dry periods, when alternative pastures are not readily available in the catchment areas.

### 3.5. The Total Economic Value Wetlands for Domestic Water Supply and Papyrus

The gross annual value of domestic water supply was estimated to be worth US$ 13.9 million (Table 9). Wetlands were the only source of water for domestic use at both household and community levels in all the study agroecological zones. The total economic value of wetlands for papyrus was assessed by estimating the value of papyrus raw materials or products that were sold for cash such as crafts and mats. The economic value of papyrus raw materials was valued with two options of either selling raw papyrus materials before processing or after value addition through mat making, which was common in all the three agroecological zones. The annual value of papyrus raw materials was estimated to be US$ 4.63 million (Table 10). Papyrus was used for wall construction, thatching houses, and making a number of craft items such as mats and chairs. Papyrus products were also sold to generate income for acquiring different household foodstuffs. The value addition to papyrus into mats was estimated to annually contribute up to US$ 11.5 million (Table 11). Responses during focus group discussions indicated that making and selling of papyrus crafts provide employment to both men and women.

### 3.6. Contribution of Wetland Nonuse Values

The estimated economic values of wetland nonuse values are presented in Table 12. The annual contribution ranged from US$ 7.06 million for water recharge and regulation to US$ 1.70 billion for flood control. The nonuse values of wetlands considered in this study were micro-climatic regulation, flood control, water regulation/discharge, habitat/refugia, and recreation. The monetary value of these services was more pronounced in the Lake Victoria crescent agroecological zone.

### 3.7. Economic Costs of Wetland Management

The wetland management costs for the financial year 2011/2012 totaled to US$ 48,668 per year (Table 13). Management costs were computed based on resources from central government funds, locally generated revenues, and salaries and allowances of the wetland staff in each agroecological zone.

### 3.8. Opportunity Costs for Limiting Access to Wetlands

The opportunity cost was estimated in the range of US$ 1.40 to 6.61 million (Table 14). The value used to estimate the foregone benefits was derived from an estimate by Karanja et al. [4], which indicated that the average benefit for maintaining biodiversity in Uganda was US$ 48.24 per hectare per year. The study considered the opportunity cost, if the current use of the wetlands was to be stopped before any modification or conversion. This would lead to foregoing foodstuffs, income, and other economic opportunities.

### 3.9. Net Economic Contribution of Wetlands to Food Security

Wetlands in the three agroecological zones provided an average net contribution of about US$ 10,491 per hectare per year (Table 15). This took into consideration the economic value derived from the various uses of the wetlands, the costs involved in the management, and the fact that the benefits of wetlands can be sustained by good management interventions. This is in line with the study by Karanja et al. [4], which estimated the net total economic value of wetlands in Pallisa District at US$ 10,861 per hectare per year, and Maclean et al. [16], which estimated benefits from Lake Bunyonyi in the range of US$ 11,200 to US$ 24,000 per hectare per year.

## 4. Discussion

### 4.1. The Economic Values of Wetlands 

#### 4.1.1. Fish Availability and Breeding/Spawning

Results show that wetlands were valued as major breeding grounds for fish. Wetlands are important for the reproduction of certain fish species like *Protopterus*, *Clarias*, *Schilbe*, *Labeo, Alestes* spp., and *Oreochromis niloticus* [17]. They are also important habitats for a number of fish species including *Clarias* spp., mudfish, *Protopterus* spp., and various *Haplochromis* spp. In addition to serving as breeding grounds, the contribution of wetlands through provision of fish is most significant for species that have respiratory systems that are adapted to seasonal flooding and can withstand reduced water levels in wetland areas such as *Clarias* spp. [18]. However, in some of the pilot areas, the spawning grounds for fish species that reproduce in wetlands are under threat as result of the ever-progressing encroachment. This justifies the need to protect wetlands for increased fisheries resources, given the fact that most fishes breed in shallow waters along wetland areas, as noted by WMD et al. [5].

Results from this study further indicate the value of wetlands through fish catch as food and source of proteins and employment to the fishing communities. During the focus group discussions, it was noted that *Clarias* sp. (catfish) are commonly harvested from wetland areas in the Kyoga plains and Southwestern farmlands agroecological zone, and provide a cheap source of animal protein and are one of the main commercial activities during the dry season when water levels of wetlands reduce, providing easy harvesting. The findings from this study indicate that the contribution of wetlands to food security through provision of fish is significant, and this is supported by other findings that in Uganda fish provides up to 50% of all animal protein [19]. The results are supported by other studies such as Akwetaireho [20], in which wetlands support livelihoods of people engaged in fishing such as fishers, boat owners, crew, and employees in fish processing factories. Wetlands are thus of importance to socioeconomic development from the fisheries sector, whose contribution in 2009 was estimated at about 2.8% of Uganda's national GDP [21]. Loss of wetlands will therefore have a significant impact on the livelihoods of local communities and will have a negative impact on the availability of fish. Benefits from fish harvest to local communities can serve as incentives for involvement in the conservation of wetlands in different areas and should therefore be enhanced.

#### 4.1.2. Crop and Livestock Farming

The economic value of wetlands through crop production was enormous. The economic contribution of wetlands through crop farming is locally and globally recognised as indicated by one of the crops valued during this study (paddy rice), regarded as a staple diet of more than half the world's population, Uganda inclusive [22]. Successes in socioeconomic development to local communities from use of wetlands for crop farming have also been reported in Ethiopia [23]. Given the current impacts of climate change on unpredictable rainfall [24], use of wetlands for crop farming will keep increasing, considering the fact that wetlands have all year round reliable moisture for crop growth. The availability of moisture and nutrients provides an opportunity for use of wetlands edges for production of different crops throughout the year, if clear guidelines for different practices are provided to minimise increased drainage of wetlands for crop farming. 

During focus group discussions, the farmers noted that over time the fertility their soils has declined, which has necessitated the use of inorganic fertilizers and pesticides, which represent a potential threat to the wetland ecosystems. These agrochemicals alter the ecological balance of wetlands and can indirectly eliminate important faunas that play a role in wetland functions and services. As noted by Dixon and Wood [23] and FAO [25], the wise use of wetland for crop farming should therefore be guided by well-defined policies and legislation to limit the amount of areas to be drained, quantities of agrochemicals to be used, in addition to use of appropriate agronomic practices. Lessons on the wise use of wetlands for crop farming are available from FAO [25] and Heimlich et al. [26]. Successes in socioeconomic development to local communities from use of wetlands for crop farming have also been reported in Ethiopia [23] and are expected to be possible in Uganda [5].

Results further show that wetlands contribute significantly to crop farming through grass mulch. Mulching helps retain the moisture, controls soil erosion, and acts as a source of organic manure in the banana plantations. Wetlands are the major remaining sources of mulch, comprising mainly of sedges including *Typha* spp. and *Cyperus* spp. [5]. During focus group discussions, it was reported that wetland grass mulch adds value to banana productivity through moisture retention and erosion control and also acts as a source of organic manure in the banana plantations. The farmers indicated that without mulch, banana yields can even reduce by 50%.

Wetlands were also valued for provision of fodder, especially during the drought periods, when alternative pastures were not readily available. Pastures from wetlands not only provided fodder but also enhanced milk production, thus contributing to food security. The importance of wetlands is also more significant due to the fact that alternative livestock feed is expensive and may not be easily affordable by most farmers in Uganda. This is more significant with the current challenges of climate change and unpredicted weather conditions [27]. However, most wetlands suffer from overgrazing. Overgrazing harm wetlands through soil compaction, removal of vegetation, and river bank or lake shore destabilization [28]. These changes in turn affect wetlands' filtering capacity, flood control capabilities, water recharge, and wildlife habitat. Other studies have identified the direct effects of livestock grazing to include the consumption of plant biomass, trampling of plants, including belowground parts and soil, nutrient inputs and bacterial contamination from dung and urine, and the introduction and dispersal of seeds and other propagules [28, 29]. Similar effects are likely to be experienced in the study wetlands where livestock grazing is the key livelihood activity. However, there is limited information on the effects of livestock grazing on water and soil quality in wetlands in the study area [15]. Studies are ongoing in Uganda that will give evidence-based information for formulation of livestock grazing guidelines.

Wetlands were valued as the most reliable water sources for livestock grazers. The importance of wetlands for livestock watering is more pronounced during dry seasons when most water sources dry up and large herds of cattle concentrate in few wetlands. However, information from focus group discussions indicated that watering livestock usually leads to grazing the livestock nearby, and when kept near streams and wetlands, they trampled river banks and lake shores, damaging vegetation resulting in increased erosion and sedimentation. This in most cases leads to soil compaction, removal of vegetation, and river bank or lake shore destabilization. It also directly adds animal waste, which most often leads to pollution. Similar impacts of livestock watering on wetlands have been noted by Belsky et al. [30], Robertson and Rowling [31], and Staton and O'Sullivan [32]. Uncontrolled grazing and watering of livestock in wetland areas also often results in increased stream turbidity, as well as increased input of nutrients and bacteria into the stream, which affects the quality of water available to downstream users. Impacts of livestock wastes contaminating streams with faecal organisms contained in the wastes, which lead to health problems for humans, have been noted by Miner et al. [33]. Such effects are very significant in Uganda, where more than 80% of the population directly use water from wetlands [5]. The impacts of livestock watering can be minimised by providing alternative livestock watering facilities as proposed by Jansen and Robertson [28], Staton and O'Sullivan [32], and Miner et al. [33].

#### 4.1.3. Papyrus

Harvesting of papyrus is one of the sustainable wetland uses of wetlands that would provide multiplier effects, in addition to direct income to papyrus harvesters and processors. This has been confirmed by studies elsewhere, such as Karanja et al. [4], Emerton et al. [11], Maclean et al. [16], and Muthuri et al. [34]. Moreover, the benefits from papyrus can motivate the users not to clear the papyrus wetlands, which would provide a relatively less degraded wetland that can provide other ecological services such as climate modulation and water purification and filtration. For example, it is known that papyrus swamps are significant sinks of carbon as they have a high net primary productivity and large amounts of detritus that can accumulate below the living mat of rhizomes and roots [16, 35–37].

#### 4.1.4. Domestic Water Supply

Another important resource provided by the wetlands in all the agroecological zones was water for domestic use. Wetlands are the main sources for the spring wells, boreholes, shallow wells, valley dams, and natural wells where local communities draw water for domestic use. As noted by Akwetaireho [20], wetland meets the daily water requirements of around 18, 885 people living close to wetlands and that about 119,249.333 litres cubic meters of water per year are collected from the watering sources scattered around the wetlands. Availability of water from wetlands enables the disadvantaged groups particularly women and children to easily access water rather than walking long distances which is an additional burden to their domestic cores.

#### 4.1.5. Nonuse Values

Wetlands were valued for the nonuse values such as micro-climatic regulation, flood control, water regulation/discharge, habitat/refugia, and recreation/tourism. Though rarely appreciated, the nonuse values contribute to the benefits that directly or indirectly play a role in food security. Thus, providing monetary figures for wetland nonuse values gives a basis for planning and decision making on the importance of leaving some wetlands intact. This is critical because the loss of most nonuse values is not easily recognised, compared to the direct resources, whose loss can be realized by lost incomes. 

It is worth to note that wetland resource utilisation activities are carried out almost exclusively by the people who live in settlements which directly border relevant wetlands. However, the benefits associated with nonuse values accrue over a much larger area, to rural and urban residents, and most of them are of public goods nature and deserve special consideration. This is recognised by different studies as one of the strong justification of leaving some wetland areas intact, with minimal disturbance as justified by Emerton et al. [11], Balmford et al. [38], Bullock and Acreman [39], and Korsgaard and Schou [40].

### 4.2. Net Economic Contribution of Wetlands to Food Security

The findings from this study indicates that if wetland resources were used unsustainably, or in a manner which reduces societal net benefits, local people's income would decline. This is likely to affect their perceived value of wetlands and would further encourage even more unsustainable levels of resource use, ultimately leading to the destruction of wetland ecosystems as observed by Korsgaard and Schou [40] and Bai et al. [41, 42]. The estimates of wetland benefits as for this study illustrate the magnitude of the economic value of wetlands in addition to their biodiversity, scientific value, climate regulation, potential tourism, social, cultural and other important wetland values. They further represent one more tool to raise awareness with decision makers about the economic year.

## 5. Conclusions and Recommendations

Results from this study provide evidence of the economic benefits derived from wetland goods and services. The study points out that many rural people's livelihoods depend directly on wetlands in addition to wetlands provision of ecosystem services. Often, these people are resource poor and they have few alternatives once the ecosystems deteriorate. It is also appreciated that the increasing human and animal populations and uncertain climatic conditions are exerting immense pressure on the different wetland resources, leading to varying levels of wetland degradation, which may lead to loss of the benefits.

One of the causes of wetland degradation is information failure, which most often is caused by lack of understanding of the values of wetlands, including the economic values. For such reasons, the protection of wetlands does not appear to be a serious alternative for resource users and cannot be advocated for by planners and policy makers. 

Findings from the study therefore hold great potential for raising awareness about the roles and economic values of wetland benefits and ecosystem services. There is need to disseminate results from this study to resource users, policy makers and implementers, and to make them recognize the economic value of wetlands and put their efforts in sustainable management of the important resources by drawing strategies to sustain the wetland benefits to society.

## Figures and Tables

**Table 1:** Wetland resources considered for economic valuation in Uganda.

Resources contribution	Southwestern farmlands	Lake Victoria crescent	Kyoga plains	Specific sites
Availability				
Fish	*✓*	*✓*	*✓*	Nakivale, Mabamba L. Nakuwa, and Limoto
Paddy rice			*✓*	Limoto, Gogonyo
Vegetables	*✓*	*✓*		Rucece, Nangabo
Yams/Taro		*✓*		Rucece, Mende, and Limoto
Maize	*✓*	*✓*	*✓*	Rucece, Nakivale, Mende, and Gogonyo
Sugar cane	*✓*	*✓*		Rucece, Mende, and Limoto
Livestock grazing	*✓*		*✓*	Nakivale, Gogonyo
Livestock watering	*✓*		*✓*	Nakivale, Gogonyo
Hunting (bush meat)		*✓*	*✓*	Mabamba, Gogonyo/Limoto
Grass for mulching	*✓*	*✓*		Rucece, Nangabo
Wild fruits and vegetables		*✓*	*✓*	Mende, Mabamba, and Gogonyo
Accessibility				
Papyrus		*✓*	*✓*	Mende, Limoto
Crafts		*✓*	*✓*	Mabamba, Gogonyo
Sand	*✓*	*✓*	*✓*	Rucece, Mabamba
Clay		*✓*	*✓*	Rucece, Mende, and Limoto
Grass for thatching	*✓*		*✓*	Nakivale, Gogonyo, and Limoto
Tourism	*✓*	*✓*	*✓*	Mabamba, L. Mburo
Services/functions				
Breeding ground for fish	*✓*	*✓*	*✓*	Nakivale, Mabamba, Limoto, and Gogonyo
Industrial/urban water supply	*✓*		*✓*	Nakivale, Rucece
Flood control	*✓*	*✓*	*✓*	Rucece, Nakivale, Mende, Mabamba, Limoto, and Gogonyo
Weather modification	*✓*	*✓*	*✓*	Rucece, Nakivale, Mende, Mabamba, Limoto, and Gogonyo
Carbon sequestration	*✓*	*✓*	*✓*	Rucece, Nakivale, Mende, Mabamba, Limoto, and Gogonyo
Domestic water supply	*✓*	*✓*	*✓*	Rucece, Nakivale, Mende, Mabamba, Limoto, and Gogonyo
Transport	*✓*	*✓*	*✓*	Nakivale, Mabamba, L. Nakuwa, and Gogonyo

**Table 2:** Monetary value of fish spawning grounds in the wetlands of Uganda.

Item	Southwestern farmlands	Lake Victoria crescent	Kyoga plains	Overall
Total spawning area (ha)*	21,459	107,833	45,339	174,631
Estimated value (US$ ha^−1^ yr^−1^)**	6.3	6.3	6.3	6.3
Total gross value per year (US$)	**134,119**	**673,956**	**283,369**	**1,091,444**

*Derived from the Uganda National Wetlands Information Systems.

**From Turpie (2000) [10] at U$ US$ 6.25  ha^−1^ yr^−1^.

**Table 3:** Per capita fish availability among the local communities in the wetlands areas of Uganda.

Item	Southwestern farmlands	Lake Victoria crescent	Kyoga plains
Number of resource users	21	17	37
Total Revenue (US$)	464,295	372,300	365,000
Human population in 2012	865,800	1,371,600	544,300
Per capita fish revenue (US$)	**0.54 **	**0.27 **	**0.67 **

**Table 4:** Monetary value of wetlands in terms of crop farming in three agro-ecological zones of Uganda.

Variable	Southwestern farmlands	Lake Victoria crescent	Kyoga plains
Major crop grown in wetlands	Maize	Vegetables (Nakatti)	Rice
Total farming area in wetlands (ha)*	932	3,065	16,335
Area of Wetland under crops (ha)	746	2,452	13,068
Yield per hectare (per season) tonnes	4	8	2
Number of harvests per year	2	3	2
Total Harvest per year (tonnes)	5,219	55,170	52,272
Price per tonne (US$)	80	60	480
Gross annual value of harvest at farm gate prices (US$ per year)	**417,536**	**3,310,200**	**25,090,560**

*Derived from the Uganda National Wetlands Information Systems.

**Table 5:** Monetary contribution of wetland grass to food security through mulching bananas in the South western Farmlands agro-ecological zone.

Variable	Isingiro district	Mbarara district	Values for southwestern farmlands
Midyear human population projections (2012)	420,200	445,600	865,800
Number of households	60,029	63,657	123,686
Total hectares of bananas (ha)	60,029	63,657	123,686
Total hectares of bananas that are mulched with wetland grass (ha)	30,014	31,829	61,843
Number of bundles of mulch applied per hectare	700	700	700
Number of times mulch is applied per year	2	2	2
Total number of bundles of mulch applied per year	42,020,000	44,560,000	86,580,000
Cost per bundle (US$)	0.10	0.10	0.10
Gross value of mulch applied (US$)	**4,202,000**	**4,456,000**	**8,658,000**

All values reflect estimates of the entire wetlands in Uganda.

**Table 6:** Monetary value of wetland pastures in three agro-ecological zones in Uganda.

Variables	Southwestern farmlands	Lake Victoria crescent	Kyoga plains	Overall
Total number cattle	330,337	114,769	136,225	581,331
% of total cattle dependant on wetlands*	10	10	10	10
Number of cattle raised in wetlands	33,034	11,477	13,623	58,133
Average value of pasture consumed per day per animal (US$)	0.20	0.20	0.20	0.20
Imputed value of pasture consumed by all animals per day (US$)	6,607	2,295	2,725	11,627
Total value of pasture consumed per year (US$)	**2,411,460**	**837,814**	**994,443**	**4,243,716**

*Estimate 10% of the cattle to directly use wetlands for grazing.

**Table 7:** Monetary value of wetlands for livestock watering in three agro-ecological zones of Uganda.

Variables	Southwestern farmlands	Lake Victoria crescent	Kyoga plains	Overall
Total number of cattle	330,337	114,769	136,225	581,331
Number of cattle obtaining water from wetlands*	33,034	11,477	13,623	58,133
Amount of water consumed per day per head of cattle (20 litre jerry cans)	2	2	2	2
Total amount of water consumed per year (20 litre jerry cans)	24,114,601	8,378,137	9,944,425	42,437,163
Cost of water per 20 litres (US$)	0.04	0.04	0.04	0.04
Gross annual value of water for livestock production (US$)	**964,584**	**335,125**	**397,777**	**1,697,487**

*Estimate 10% of the cattle to directly use wetlands for watering.

**Table 8:** Gross monetary value addition from wetlands through milk production in Uganda.

Variables	Southwestern farmlands	Lake Victoria crescent	Kyoga plains	Overall
Total number of cattle	330,337	114,769	136,225	581,331
Number of milked cows*	56,100	22,290	12,600	90,990
Average milk production per cow per week (litres)*	7.1	25.6	5.3	19.0
Total annual milk production (litres)	20,566,260	29,672,448	3,472,560	89,779,833
Percentage attributed to wetlands	10	10	10	10
Milk production assessed to wetlands (litres)	2,056,626	2,967,245	347,256	8,977,983
Price of milk (US$)	0.13	0.13	0.15	0.14
Gross value of milk production per annum (US$)	**257,078 **	** 398,798 **	** 51,394 **	**1,219,210 **

*Derived from MAAIF and UBOS, 2009 [15].

**Table 9:** The gross annual monetary value of wetlands for domestic water supply in three agro-ecological zones in Uganda.

Variable	Southwestern farmlands	Lake Victoria crescent	Kyoga plains	Overall
Midyear human population projections (2012)*	865,800	1,371,600	544,300	2,781,700
Number of households	123,686	195,943	77,757	397,386
Households dependant on wetlands for water supply**	98,949	156,754	62,206	317,909
Average use of water (20 litre jerrycans)	3	3	3	3
Water use for all the households per year (m^3^)	2,166,974	3,432,919	1,362,305	6,962,198
Market price per m^3^ (US$)***	2	2	2	2
Gross annual value of water for domestic consumption (US$)	**4,333,947**	**6,865,838**	**2,724,610**	**13,924,395**

*Human population projections were based on midyear values of 2012.

**Estimated at 80% from WMD et al. (2009) [5].

***Computed based on the price of a 20 litre jerrycan at US$ 0.04.

**Table 10:** Monetary value of papyrus raw materials without value addition in three wetland agro-ecological zones in Uganda.

Variables	Southwestern farmlands	Lake Victoria crescent	Kyoga plains	Overall
Total area under papyrus (ha)*	12,713	20,751	32,095	65,559
Productivity per hectare (head loads)	400	400	400	400
Total productivity per annum (head loads)	5,085,200	8,300,400	12,838,000	26,223,600
Price per head load (US$)	0.17	0.20	0.16	0.18
Total gross value of papyrus production (US$)	** 864,484 **	**1,660,080 **	**2,054,080 **	**4,632,836 **

*Derived from Uganda National Wetlands Information Systems.

**Table 11:** The economic value of papyrus after value addition through mat making.

Variables	Southwestern farmlands	Lake Victoria crescent	Kyoga plains	Overall
Total productivity (head loads)	5,085,200	8,300,400	12,838,000	26,223,600
Number of head loads converted into 2.5 m × 3.5 m mats (SW Farmlands: 75%, LVic. Crescent: 80%, Kyoga Plains: 65%)	3,813,900	6,640,320	8,344,700	19,230,640
Number of mats produced (1 : 2 conversion ratio)	7,627,800	13,280,640	16,689,400	38,461,280
Price per mat (US$)	0.40	0.40	0.40	0.40
Gross value of papyrus mats produced (US$)	3,051,120	5,312,256	6,675,760	15,384,512
Cost of processing inputs (US$)	762,780	1,328,064	1,668,940	3,846,128
Gross value addition (US$)	**2,288,340**	**3,984,192**	**5,006,820**	**11,538,384**

**Table 12:** Monetary contribution of wetland non-use values in three agro-ecological zones of Uganda.

Variable	Monetary value US$ ha^−1^ yr^−1^	Southwestern farmlands	Lake Victoria crescent	Kyoga plains	Overall
Area (ha)		29,155	137,125	68,932	235,212
Microclimatic regulation	265	7,726,075	36,338,125	18,266,980	62,331,180
Flood control	7,240	211,082,200	992,785,000	499,067,680	1,702,934,880
Water regulation/recharge	30	874,650	4,113,750	2,067,960	7,056,360
Habitat/refugia	439	12,799,045	60,197,875	30,261,148	103,258,068
Recreation/aesthetic	491	14,315,105	67,328,375	33,845,612	115,489,092
Cultural	1,761	51,341,955	241,477,125	121,389,252	414,208,332

*Values derived from Karanja et al. (2001) [4].

**Table 13:** Costs for wetland management and conservation in three agro-ecological zones of Uganda (Data for 2011/2012).

Item	Southwestern farmlands	Lake Victoria crescent	Kyoga plains	Overall
Central government funding (US$)	8,040	4,840	5,988	18,868
Local revenue (US$)	1,400	3,600	1,400	6,400
Salary and allowances (US$)	9,600	5,760	8,040	23,400
Total management costs (US$)	** 19,040 **	** 14,200 **	** 15,428 **	** 48,668 **

**Table 14:** Opportunity costs of limiting community access to wetlands in three agro-ecological zones in Uganda.

Variable	Southwestern farmlands	Lake Victoria crescent	Kyoga plains
Area (ha)	29,110	137,125	68,932
Opportunity cost (US$ ha^−1^ yr^−1^)	48.24	48.24	48.24
Total opportunity cost (US$)*	** 1,404,266 **	**6,614,910 **	**3,325,280 **

*Based on national average of US$ 48.24 ha^−1 ^derived from Karanja et al. 2001 [4].

**Table 15:** Summary of total economic contribution of wetlands in three agro-ecological zones of Uganda.

Resource contribution (US$)	Southwestern farmlands	Lake Victoria crescent	Kyoga plains
Availability			
Fish breeding/spawning	134,119	673,956	283,369
Fish production	464,295	372,300	365,000
Crop farming	417,536	3,310,200	25,090,560
Livestock grazing/pastures	2,411,460	837,814	994,443
Livestock watering	19,291,681	6,702,510	7,955,540
Value added through milk production	7,717	2,681	3,182
Wetland grass for mulching	4,202,000	4,456,000	8,658,000
Accessibility			
Papyrus	864,484	1,660,080	2,054,080
Papyrus crafts	2,288,340	3,984,192	5,006,820
Services/functions			
Domestic water supply	4,333,947	6,865,838	2,724,610
Nonuse values	298,139,030	1,402,240,250	704,898,632
Total economic value to food availability	** 26,928,808 **	** 16,355,461 **	** 43,350,094 **
Total economic value to food accessibility	** 3,152,824 **	** 5,644,272 **	** 7,060,900 **
Total economic value through services and functions	** 302,472,977 **	** 1,409,106,088 **	** 707,623,242 **
*Total economic value of wetlands to food security *	**332,554,609**	**1,431,105,821**	**758,034,236**
Costs of management and maintenance of wetlands			
Management costs	19,040	14,200	15,428
Opportunity costs	1,404,266	6,614,910	3,325,280
*Total economic cost to maintain the wetlands *	**1,423,306**	**6,629,110**	**3,340,708**
* Net economic value of wetlands for food security *	**331,131,303**	**1,424,476,711**	**754,693,528**
*Net benefits per hectare per year (US$) *	**11,358**	**10,388**	**10,948**
